# Prochlorperazine-Induced Hemidystonia Mimicking Acute Stroke

**DOI:** 10.5811/westjem.2015.4.26003

**Published:** 2015-07-02

**Authors:** Zlatan Coralic, Anthony S. Kim, David R. Vinson

**Affiliations:** * University of California, San Francisco, Departments of Pharmacy and Emergency Medicine, San Francisco, California; † University of California, San Francisco, Department of Neurology, San Francisco, California; ‡ Kaiser Permanente Medical Center, Department of Emergency Medicine, Sacramento, California; § Kaiser Permanente Division of Research, Oakland, California

## Abstract

Prochlorperazine is frequently used in the treatment of refractory nausea and migraines. Known side effects include extrapyramidal symptoms such as akathisia and dystonia. We report a pregnant patient taking prochlorperazine for hyperemesis gravidarum who developed hemidystonia, which triggered an acute code stroke response from prehospital, emergency medicine and neurology providers. We suspect this report to be the first case of prochlorperazine-induced hemidystonia as a stroke mimic.

## INTRODUCTION

Stroke is a leading cause of death and permanent disability among adults in the developed world.^1^ Intravenous fibrinolytics improve outcomes in patients with ischemic stroke.^2^ However, the efficacy of fibrinolytic therapy is highly time-dependent, which has driven efforts to reduce the time to diagnosis, imaging, and medication administration.^3^–^4^

In the push to administer fibrinolytic therapy, healthcare providers must not circumvent a thorough assessment of the contraindications to therapy as well the potential for a stroke mimic.^5^ The search for stroke mimics might be especially important when off-label use of fibrinolytic stroke therapy in pregnancy is being considered.^6^

We report a case of a pregnant woman who presented to the emergency department (ED) with recent-onset hemiplegia that was initially thought to be an acute ischemic stroke. Only when further signs developed that suggested a dystonic reaction and a culpable medication was identified did we entertain the possibility of a stroke mimic. The resolution of her neurological symptoms shortly after administration of diphenhydramine confirmed the most likely diagnosis of dystonia. We believe this is the first case of prochlorperazine-induced hemidystonia as a stroke mimic reported in the literature.

## NARRATIVE

Our ED received a stroke activation from the field. The initial report was of a 32-year-old woman with unilateral stroke-like symptoms. This G3P1 10-week pregnant woman arrived to the ED via ambulance complaining of sudden-onset slurred speech and left chest, shoulder, and neck pain that began 40 minutes prior to ED arrival. This was followed by severe left arm and leg weakness and paresthesias. Her past medical history included a laparoscopic appendectomy four days prior and hyperemesis gravidarum during this pregnancy.

Her vital signs were blood pressure 114/79mmHg, heart rate 106 beats/min, respiratory rate 25 breaths/min, temperature 36.9°C, and oxygen saturation 100% on room air. On exam, the patient was alert and oriented with intermittent slurred speech. She was repeatedly crying “help me,” but was directable and able to follow commands. Her face was symmetric and her tongue was midline with a small amount of accumulated saliva. The patient’s left arm and leg were described as being weak and she was unable to lift them during exam. The rest of the motor and sensory exam were normal.

As all suspected strokes are co-managed with the admitting neurology team at our institution, an in-house “code stroke” was activated, and head computed tomography (CT) was ordered per hospital stroke protocol. Subsequent laboratory values including chemistry panel, liver function tests, coagulation panel, and complete blood count were unremarkable.

Within five minutes the admitting neurology resident arrived and agreed with the need for emergent head CT. While the patient was being prepared for the scan, we observed that her tongue was beginning to protrude and roll repetitively, raising suspicion for extrapyramidal symptoms (EPS). Upon reviewing the bedside chart, we noted that the patient’s only medications were ondansetron and prochlorperazine. When additional medication history was elicited, we discovered that prochlorperazine was a new medication that had been initiated one day before presentation. The patient reported taking a total of four 10mg doses of prochlorperazine by mouth, with the last dose taken one hour prior to symptom onset. The patient’s weight at the time of the visit was 67kg.

We administered a 50mg dose of intravenous diphenhydramine and the patient’s motor symptoms rapidly resolved. However, she still complained of residual left upper and lower extremity weakness, although her neurological exam was now normal, with a National Institute of Health Stroke Scale of 0. On serial exams her strength gradually improved, but per patient, her strength did not immediately return to baseline. She was immediately taken for magnetic resonance imaging, which revealed no evidence of an acute stroke. She was admitted to the hospital for observation and during the night experienced a second episode of dystonia that again responded to 50mg of intravenous diphenhydramine. All of the patient’s symptoms resolved by the next day and she was discharged from the hospital without any long-term sequelae. Seven months later the patient had an uneventful delivery of a healthy term baby.

## DISCUSSION

Stroke mimics are common in emergency medicine. In a single-center study of consecutive patients presenting to an urban teaching ED with stroke-like symptoms, 31% (109/350) of patients with suspected stroke were later determined to have a stroke mimic.^7^ The most common mimics were seizures, sepsis, and toxic/metabolic causes. The authors did not elaborate on toxic causes or differentiate medication effects. In another single-center review, acute ischemic stroke misdiagnosis occurred in 10.4% (56/539) of presentations with conversion disorder, migraines, and seizures being the most common mimics.^8^ Dystonic reactions have been described as sequelae of acute stroke and brain injury,^9^ but hemidystonic drug reactions mimicking stroke have not been reported.

Our patient experienced an EPS, specifically dystonia, which was precipitated by the appropriate use of prochlorperazine for nausea. Dystonia is defined as a disorder causing involuntary muscle contractions, repetitive movements, or contracted postures. It is important to note that our patient reported pain preceding and during the dystonic reaction. Hemorrhagic and less likely ischemic stroke may present with a severe headache, but neither stroke should cause other body pain. In contrast, one of the most common complaints in patients with dystonia is pain in the affected region.^10^ However, during stroke evaluation of a patient with hemiplegia and limited history in the acute setting, the presence or absence of pain may be too non-specific to rule out a stroke or other emergent vasculopathy.

Diagnostic momentum may have been at play in this case.^11^ When the emergency responders first encountered the patient in the field they administered the Cincinnati Stroke Scale, which was abnormal on two parameters (slurred speech and left arm paralysis).^12^ The emergency responders appropriately activated the stroke response per city protocol. In the ED, these findings were confirmed with a rapid history and neurological exam. To complicate the initial evaluation, the patient answered at first that she was not taking any medications, undoubtedly due to the distressing situation she was in. Further, additional stress was placed on the care team since the patient was pregnant and guidelines for treating pregnant women with stroke are limited.^6^ The incoming neurology resident joined a “moving train” when they arrived in the ED just as the patient was being prepared for the scan. The limited available history and the patient’s lateralized decreased movement were enough to persuade the resident to agree with the provisional diagnosis and the need for an urgent scan. As the bed was unlocked for transport to the scanner, the bedside emergency medicine clinical pharmacist pointed out to the team the repetitively protruding tongue movements that had not been apparent initially. This observation led to additional history taking and medication verification with the patient’s husband. It was only at this time that the momentum was paused and team consensus was reached to attempt a quick trial of a reversal agent.

The most significant potential harm for a patient misdiagnosed with an ischemic stroke is receiving intravenous thrombolytics developing an intracranial hemorrhage. While a single-center study found a favorable safety profile for administration of thrombolytics to patients with stroke mimics,^8^ our patient had an additional level of complexity due to pregnancy. Studies describing the use of thrombolytics in pregnant patients are limited to individual case reports with some concern about effects both to the mother and the fetus.^6^ Any discussion about risks and benefits of thrombolytics in pregnancy remains speculative at this time.

While there is no perfect method to establish the causality of an adverse drug event, the commonly used Naranjo criteria estimate the probability of medication causing an adverse event.^13^ The Naranjo score for our case was seven, which would classify this event as a probable adverse drug reaction.

Emergency clinicians should be aware of EPS in patients taking antidopaminergic agents. Other than the more common presentations of EPS, such as acute akathisia and dystonia/torticollis that may be seen from single doses of antidopaminergics (e.g., metoclopramide, prochlorperazine) given in the ED, emergent acute airway obstruction due to supraglottic dystonia has also been reported.^14^ In addition to obtaining a list of medications that may contraindicate thrombolytic use, emergency clinicians need to remain vigilant of antidopaminergic agents that may mimic common stroke-like symptoms, especially in the elderly, who may be more sensitive to these agents. Empiric administration of anticholingeric agents in every patient with stroke-like symptoms is certainly not warranted and may confound subsequent neurological exams. However, if antidopimenergics are on the medication list during an acute evaluation of stroke, clinicians should broaden the differential to include the possibility of EPS as part of the rapid initial evaluation of a patient with suspected stroke.

## References

[1] Rothwell PM, Coull AJ, Silver LE (2005). Population-based study of event-rate, incidence, case fatality, and mortality for all acute vascular events in all arterial territories (Oxford Vascular Study). Lancet.

[2] Emberson J, Lees KR, Lyden P (2014). Effect of treatment delay, age, and stroke severity on the effects of intravenous thrombolysis with alteplase for acute ischaemic stroke: a meta-analysis of individual patient data from randomised trials. Lancet.

[3] Ebinger M, Kunz A, Wendt M (2015). Effects of Golden Hour Thrombolysis: A Prehospital Acute Neurological Treatment and Optimization of Medical Care in Stroke (PHANTOM-S) Substudy. JAMA Neurol.

[4] Van Schaik SM, Van der Veen B, Van den Berg-Vos RM (2014). Achieving a door-to-needle time of 25 minutes in thrombolysis for acute ischemic stroke: a quality improvement project. J Stroke Cerebrovasc Dis.

[5] Zinkstok SM, Engelter ST, Gensicke H (2013). Safety of thrombolysis in stroke mimics: results from a multicenter cohort study. Stroke.

[6] Selim MH, Molina CA (2013). The use of tissue plasminogen-activator in pregnancy: a taboo treatment or a time to think out of the box. Stroke.

[7] Hand PJ, Kwan J, Lindley RI (2006). Distinguishing between stroke and mimic at the bedside: the brain attack study. Stroke.

[8] Tsivgoulis G, Alexandrov AV, Chang J (2011). Safety and outcomes of intravenous thrombolysis in stroke mimics: a 6-year, single-care center study and a pooled analysis of reported series. Stroke.

[9] Alarcon F, Zijlmans JC, Duenas G (2004). Post-stroke movement disorders: report of 56 patients. J Neurol Neurosurg Psychiatry.

[10] Comella C, Bhatia K (2015). An international survey of patients with cervical dystonia. J Neurol.

[11] Croskerry P (2003). The importance of cognitive errors in diagnosis and strategies to minimize them. Acad Med.

[12] You JS, Chung SP, Chung HS (2013). Predictive value of the Cincinnati Prehospital Stroke Scale for identifying thrombolytic candidates in acute ischemic stroke. Am J Emerg Med.

[13] Naranjo CA, Busto U, Sellers EM (1981). A method for estimating the probability of adverse drug reactions. Clin Pharmacol Ther.

[14] Newton-John H (1988). Acute upper airway obstruction due to supraglottic dystonia induced by a neuroleptic. BMJ.

